# Using simultaneous scanpath visualization to investigate the relationship between accuracy and eye movement during medical image interpretation

**DOI:** 10.16910/jemr.10.5.11

**Published:** 2018-02-24

**Authors:** Alan Davies, Simon Harper, Markel Vigo, Caroline Jay

**Affiliations:** University of Manchester, UK

**Keywords:** Eye movement, eye tracking, visualization, electrocardiogram, ECG, EKG

## Abstract

In this paper, we explore how a number of novel methods for visualizing and analyzing differences in eye-tracking data, including scanpath length, Levenshtein distance, and visual transition frequency, can help to elucidate the methods clinicians use for interpreting 12-lead electrocardiograms (ECGs). Visualizing the differences between multiple participants' scanpaths simultaneously allowed us to answer questions including: do clinicians fixate randomly on the ECG, or do they apply a systematic approach?; is there a relationship between interpretation accuracy and visual behavior? Results indicate that practitioners have very different visual search strategies. Clinicians who incorrectly interpret the image have greater scanpath variability than those who correctly interpret it, indicating that differences between practitioners in terms of accuracy are reflected in different eye-movement behaviors. The variation across practitioners is likely to be the result of differential training, clinical role and expertise.

## Introduction

Scanpath analysis -- examination of the sequence in which people fixate on different parts of a stimulus -- is widely used in eye-tracking research ( 1 ). Scanpaths can be considered in terms of the sequence of AOIs (Areas Of Interest defined by the researcher) that a participant visits, which can be compared with string metrics such as the Levenshtein distance, or in terms of the spatial positions/alignment of fixations (vector sequence alignment). Methods such as vector strings can also include temporal aspects like fixation duration and saccadic amplitude ( 1 ). Scanpath analysis attempts to provide insight into the cognitive processes of users interacting with a visual stimulus, as eye movements have been linked to decision making ( 2 ).

A basic method for enabling the visual comparison of scanpaths is the gaze plot, which displays all fixation data for a participant or set of participants over the stimulus. While this is comprehensive in the information it supplies, it can quickly become difficult to interpret, due to the complexity of gaze data.

Here we present a method for scanpath analysis, which combines the Levenshtein distance and other visualization methods to produce summary data that can be simultaneously visualized for multiple participants in a simple matrix form. This allows us to query the data visually, and identify similarities and differences between participants at a glance.

The particular case we examine is clinician interpretation of elctrocardiogram (ECG) images. Eye tracking has been used to explore how humans interact with data in a variety of medical domains, most notably in radiology ( 3, 4, 5 ).

This work has primarily provided a qualitative interpretation of the diagnostic process, however. Here, we apply our methods to quantitatively analyze clinicians’ visual behavior in the medical sub-domain of electrocardiology. This field particularly lends itself to scanpath analysis, as electrocardiogram (ECG) data consists of signals from 12 sources, which are presented in different equal-sized areas on a single output. These areas naturally form pre-existing “Areas of Interest” (AOIs) which can be interrogated for quantitative analysis. Here we examine the scanpaths of clinicians as they attempt to interpret ECGs. To do this we consider the transition behavior between the leads by determining and visualizing the Levenshtein distance. We do this to identify any systematic and consistent approaches taken to interpretation that are modelled by visual behavior, especially to determine if there are differences in this behavior that are attributed to the correct or incorrect interpretation of the ECG.

### Electrocardiology

The electrical activity generated by the myocardium (heart) can be represented in graphical form by the 12lead electrocardiogram (ECG) ( 6 ).

**Figure 1.**
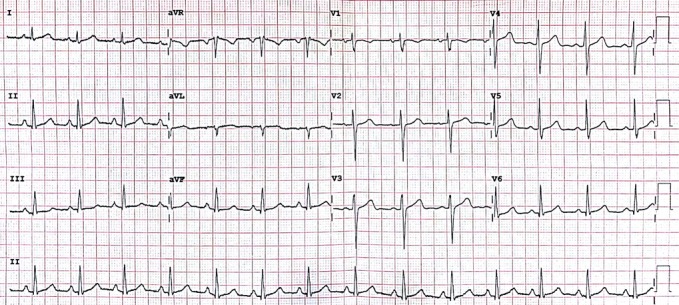
A “normal” 12-lead ECG

The ECG is one of the most commonly used medical tests and is carried out in a large variety of clinical environments ( 6 ). This is primarily down to its low cost and availability. The electrical output is displayed as a waveform that is composed of various waves (P, Q, R, S, T, U), intervals (PR, QT, QRS) and the ST segment that represent the depolarization and repolarization of the constituent components of the cardiac conduction system ( 6, 7 ). The waveform is displayed on a grid (Figure 1), where time in seconds is represented on the x-axis and amplitude in millivolts on the y-axis ( 8 ).

The different “leads” are displayed as 12 equally sized regions on the graph that are labelled. The leads labelled I, II, III, aVR, aVL, aVF display activity “viewed” from the coronal/frontal plane. Leads V1 to V6 view the transverse plane. The waveforms are presented differently in the different leads due to the direction of the electrical impulse relative to the poles of the electrodes that are attached to the surface of the patient ( 9 ).

Interpretation of the ECG is considered a complicated task and is carried out by a number of healthcare practitioners, including doctors, nurses and allied health professionals, paramedics and specially trained cardiac physiologists/technicians. Failing to make a correct interpretation of the underlying medical conditions presented on the ECG can lead to inappropriate/incorrect or no treatment being given, leading in some cases to injury and even death ( 10 ). Despite ongoing improvements in the field of automated ECG interpretation, humans are still more reliable ( 11 ) and remain the end point in interpretation as automated solutions are frequently inaccurate ( 12 ). The study presented in this paper represents a subsection of wider exploratory work related to the visual behavior of humans interpreting ECGs using eye-tracking technology. Understanding this process could provide essential information for improving automated interpretation software. This work synthesizes varied disciplines, including computer science, medicine and psychology. The initial stage reported in this paper concerns visual analysis of eye-movement data for hypothesis generation.

### Scanpath analysis techniques

Similarity between two or more scanpaths can be estimated by applying scanpath comparison measures ( 1 ).

The scanpath can also be formed from a set of locations represented by the order in which the AOI is visited (in computing terms, a string). One such method for the calculation of differences between two string sequences is the Levenshtein distance. It works by imposing a cost (penalty) for each operation (insertion, deletion and substitution) carried out to transform one string into another, where they both contain the same tokens in the same sequence ( 13 ). The Levenshtein distance is still one of most frequently used methods applied to scanpath comparison ( 1, 14 ) with applications spanning multiple domains, including the scanning of websites ( 15 ) and reasoning about others mental status ( 16 ).

Other string edit distances also exist, including the Damerau-Levenshtein distance, Hamming distance and Longest Common Subsequence (LCS) technique ( 14 ). The initial Levenshtein distance has been adapted and improved. In one such example, Galgani et al. ( 17 ) augmented the Levenshtein distance with the NeedlemanWunsch approach. This allows for the definition of custom defined cost functions. This approach was applied to improve evaluation and diagnostic methods for classification of attention disorders ( 17 ). Alternative methods for the visualization of scanpaths include the Voronoi method, a spatial method comparable to clustering fixations ( 18 ). Dotplots have also been used to visualize scanpath similarities for the purpose of validation and exploration ( 19 ).

In this work we apply visualization methods to explore similarities and differences between participants’ scanpaths as they carry out an ECG interpretation task.

## Methods

### Participants

Thirty one participants (males=8, females= 23) whose clinical role includes regularly interpreting ECGs took part in the study. Participants had an average of 9 years’ experience in interpreting ECGs (range=29). Participants were recruited from 3 hospitals in the north-west of England. They belonged to 3 main professional categories: cardiac physiologists/technicians (n=19), doctors/nurses (n=7) and students (n=5).

### Stimuli

Participants viewed eleven 12-lead ECGs taken from open access on-line libraries ( http://lifeinthefastlane.com/ecg-library/ and www.emedu.org/ecg\_lib/index.htm) and displayed in a random order on a computer screen. The ECGs represented a selection of conditions that would be encountered in clinical and training scenarios:

•Anterolateral STEMI (ST-segment elevation myocardial infarction)

•Atrial Flutter

•Hyperkalaemia

•Torsades de pointes (polymorphic ventricular tachycardia)

•Wolff-Parkinson-White syndrome (WPW)

•Ventricular tachycardia (VT)

•Left bundle branch block (LBBB)

•Normal sinus rhythm (NSR)

•Supra-ventricular tachycardia (SVT)

•Ventricular paced rhythm

•Sinus tachycardia

### Procedure

**Figure 2.**
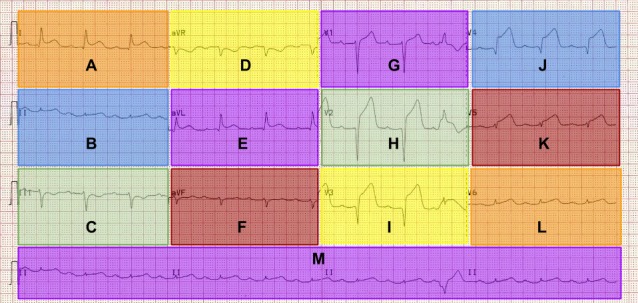
AOIs mapped onto ECG leads. Labelled A-M

The ECGs were presented in random sequence. No time limit was imposed, allowing participants to take as much time as they needed to reach an interpretation. Their interpretation, which was spoken aloud, was recorded with a voice recorder. Tobii X2-60 and 1750 eyetrackers were used to capture gaze-data as participants viewed the ECGs. Areas of interest (AOIs) labelled A-M were generated with Tobii studio software (V.1.2) for each of the 12-leads and the rhythm strip, which is an existing lead that is displayed for a longer time period at the bottom of the image (see Figure 2). Following the study, participants’ interpretations were rated as correct or incorrect for each ECG by two expert interpreters. The full stimuli, protocol, data and analysis code are available from our data repository( http://iamdata.cs.manchester.ac.uk/investigations/12).

## Analysis

Many studies focus on determining the similarity of eye-movements across participants ( 20 ). Standard techniques, including heat/focus maps and gaze-plots are limited, as they often fail to properly display the sequential/temporal nature of these eye-movements ( 20 ), or allow for subject comparisons with multiple participants, without the introduction of the excessive visual complexity. Gazeplots display the fixation sequence superimposed on a stimulus, and therefore potentially allow a comparison between participants to be made visually. Gazeplots can, however, become overly complicated and even meaningless with large group comparisons (or even just a small subset of participants).

Scanpaths can be represented as a set of tokens or characters, referred to as “strings”. The string contains the sequence of AOIs visited by a participant. This can be seen in an example from two participants in this study who viewed the anterolateral STEMI ECG.

P_25_ = {M,M,I,I,M,G,G,E,E,B,A,A,M,M,I,I}

P_19_ = {H,E,D,D,E,H,H,G,G,G,I,F,F,F,D}

Differences in fixation duration, fixation count, or the total amount of time spent viewing a particular AOI can be used to identify participant similarity. This does not, however, capture the similarity in the way participants visually transition around the ECG. This is a potentially important factor, as cross referencing different leads of the ECG is crucial to the correct interpretation of certain conditions, such as heart attacks. To examine these similarities we apply the Levenshtein distance to compute the distance (measure of similarity) of each participant with all the other participants in the study or sub-group. The distance is determined by the minimum number of insertion, deletion and substitution operations required to transform one string into another ( 1, 13 ).

When viewing the scanpath lengths for each stimulus we truncate (collapse) the scanpath to remove consecutive tokens. This is done to focus on the sequence of AOIs visited, essentially removing fixation frequency, i.e. a scanpath string consisting of *{M,M,M,B,B,A,B,C}* would become *{M,B,A,B,C}*. Unless stated specifically the results represent the un-truncated scanpaths.

The scanpath analysis reported here focuses primarily on the anterolateral STEMI ECG, as the identification of a \quotes{heart attack} is a critical skill that is taught to ECG interpreters of all levels, as opposed to specialists (cardiologists). In order to identify the STEMI, one needs to first identify ST-segment elevation, then rule out other causes (i.e. pericarditis, pacemaker, bundle branch block) before finally identifying the leads affected ( 9 ). The pattern of ST elevation in certain leads identifies what type of STEMI it is. Table 1 shows the portion of the heart that the changes reflect. For example ST elevation in the inferior leads (II, III and aVF) would indicate an inferior STEMI. There can also be combinations of areas affected. The anterolateral STEMI would involve ST elevation in both the lateral and anterior leads. In order to make the correct interpretation, ST elevation needs to be identified in each relevant lead. This makes the STEMI stimuli a good starting point for exploratory analysis as with other conditions, the salient features can be identified in different leads on an individual basis or systematically.

**Table 1.** ECG leads and portion of heart effected.

**STEMI leads**	**Myocardial area**
II, III, aVF	Inferior
I, aVL, V5, V6	Lateral
V1, V2, V3, V4	Anterior

To this end each of the participants’ scanpaths were compared against all the other participants’ for this stimulus and the results were displayed using a matrix to allow for rapid visual comparison. The darker the matrix cell the greater the difference between compared scanpaths; conversely the lighter the cell the greater the scanpath similarity. This method of visualization also makes it easier to spot outliers and make multiple comparisons simultaneously. In addition to this, we were interested in the specific areas of the stimulus that were fixated the most. It was hypothesized that these areas may be different from the top down researcher-defined AOIs that were mapped onto each ECG lead. This is because we know from ECG training texts that in order to interpret the ECG correctly one needs to focus on specific parts of the ECG waveform (the various waves, intervals and segments). In order to define these areas in a nonarbitrary data-driven way we use the DBSCAN clustering algorithm (Density-based spatial clustering of applications with noise) ( 21 ). This allowed us to cluster fixations and then subsequently determine the smallest radius for what is termed a “core point” (threshold for the number of points in a given radius to be included in a core point). We use this value to inform the cell size for a grid (minimum cell dimension = core point diameter). As the stimulus is rectangular, the smallest cell dimension is used to determine the width of the cell. This allows cells to be rectangular, in order to increase coverage of the stimulus. We are then able to detect fixations in each grid cell and generate heat maps based on these values. As the cell sizes for each stimulus are the same, we can then produce heatmaps for the correct and incorrect groups for each ECG and directly compare differences between cells to quantify how similar or different they are as well as using them to identify key areas of attention. All statistical analysis was carried out using the R project for statistical computing, version 3.3.2. ( 22 ), with α < 0.05. Mann-Whitney U tests were used to compare groups with non-parametric data. We also demonstrate the utility of web diagrams for analyzing scanpath length, and chord diagrams for showing differences in transition behavior.

## Results

We present the results in terms of the scanpath lengths and differences between scanpaths across all stimuli using the Levenshtein distance. We then focus on the anterolateral STEMI stimuli, looking at scanpath similarities for the correct and incorrect interpretation groups. Finally we look at distribution of attention using datadriven heatmaps, and differences in visual transition behavior between the salient leads.

**Figure 3.**
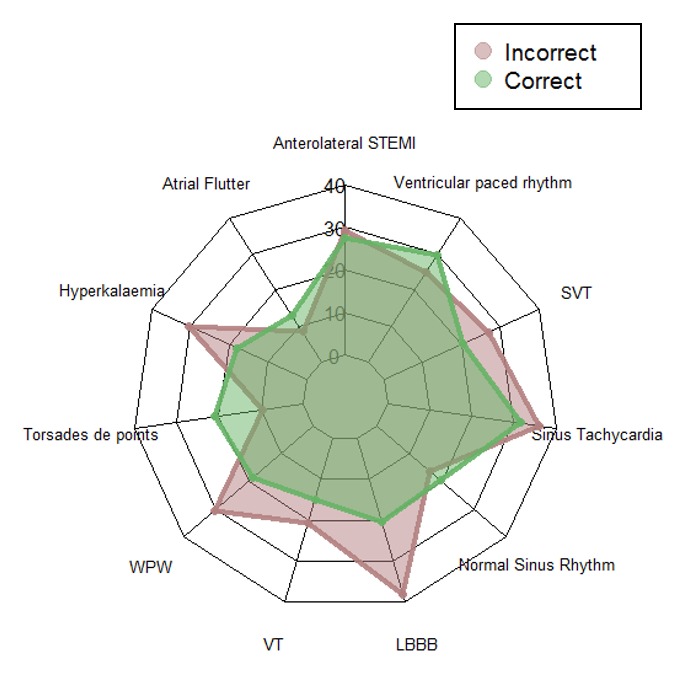
Average scanpath lengths for each stimulus for correct and incorrect groups

The aggregated scanpath lengths (with truncation) representing the scanpath as the sequence of AOIs visited are shown in the web diagram in Figure 3 for both groups for each ECG. The average length of the scanpaths across all stimuli for the combined groups was 23 AOIs (SD= 18.25, Mo=9, range=134).

**Figure 4.**
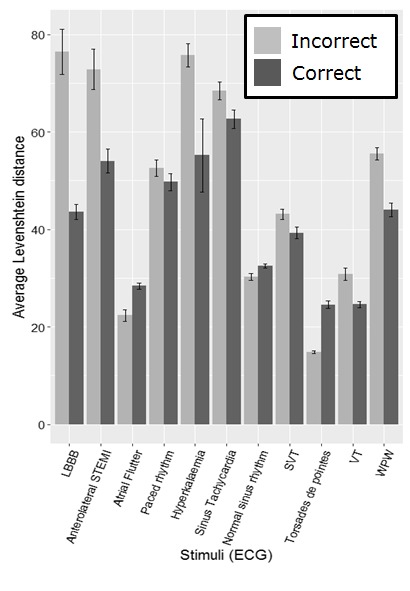
The average Levenshtein distance for both groups for each ECG (errorbars represent the SE)

Figure 4 shows the average Levenshtein distance per group for each ECG. As the number of participants making correct and incorrect interpretations varies considerably across the different ECGs (Table 2), using standard statistical comparisons is problematic in all but one case. It is necessary to group participants into correct and incorrect interpretation groups per stimulus on a post hoc basis, as they may get a certain ECG right and another wrong and vice versa, making it impossible to assign them to groups prior to beginning the task. The Anterolateral STEMI (heart attack) has fairly evenly sized groups making comparison possible. We compared the average Levenshtein distance for the correct and incorrect groups using a Mann-Whitney U test for this stimulus, which highlights a significant difference (W = 21284, p = .004), with the incorrect group having a larger Levenshtein distance on average (M=86, SD=102.63) than the correct group (M=46, SD=12.53).

**Table 2.** The number of participants making correct and incorrect interpretations per ECG.

**Stimuli (ECG)**	**Correct (n)**	**Incorrect (n)**
Anterolateral STEMI	16	14
Atrial Flutter	26	5
Hyperkalaemia	2	30
Torsades de pointes	5	27
WPW	13	18
VT	27	5
LBBB	24	8
NSR	24	7
SVT	10	21
Ventricular paced	9	22
Sinus tachycardia	12	20

**Figure 5.**
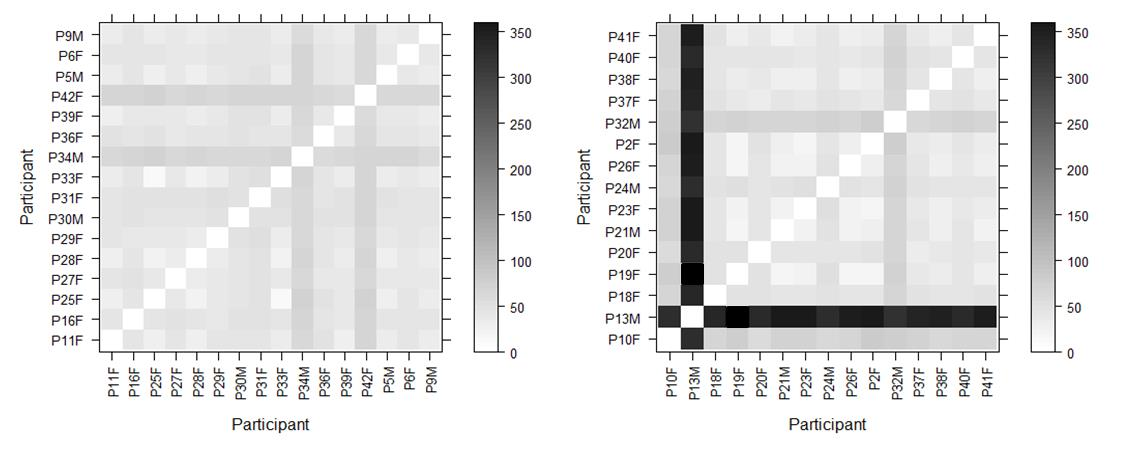
Levenshtein distance plots for correct (left) and incorrect (right) groups for the anterolateral STEMI ECG.

**Figure 6.**
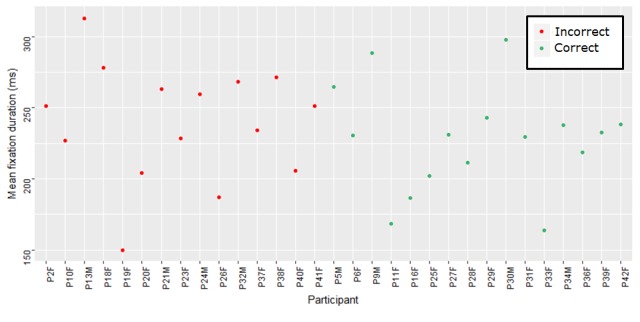
Average fixation duration for each participant for anterolateral STEMI ECG by group

Matrix visualizations (Figure 5) are used to compare each participant against every other participant in the group (correct or incorrect). The darker the cell, the greater the distance, meaning that the compared scanpaths are less similar. The plots are normalized by the maximum Levenshtein distance to aid visual comparison. Participant 13 (P13M, a student cardiac physiologist) in the incorrect group has a very different scanpath to all of the other participants. This participant also has the longest individual scanpath length (377) and the longest average fixation duration (M=312.97, SD=384.86). Figure 6 shows the average fixation duration per participant for each group for the STEMI ECG.

**Figure 7.**
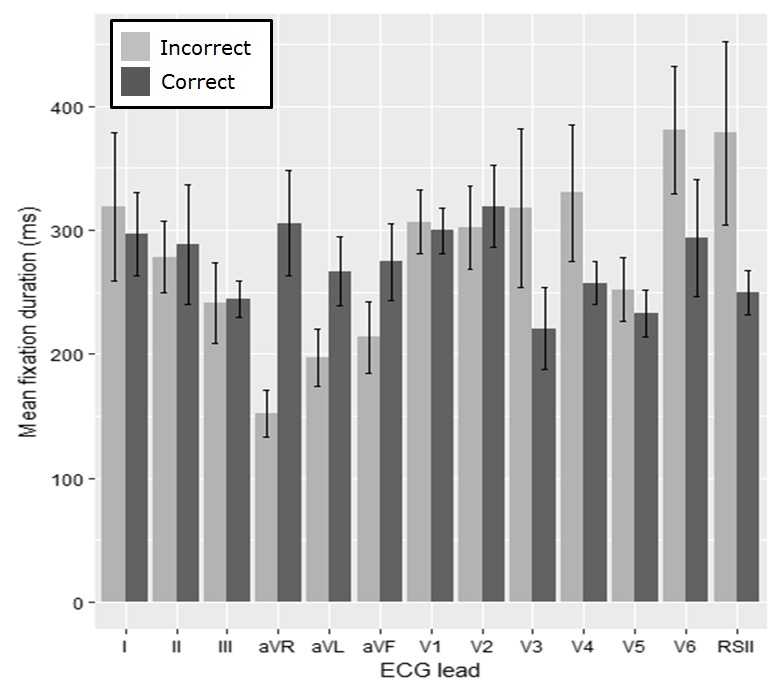
Average fixation duration for both groups, per lead (errorbars represent the SE)

The average fixation duration for each lead of the ECG for the anterolateral STEMI (Figure 7) is then examined. For the fixation duration we apply pairwise comparisons with Bonferroni correction (α = 0.004). A significant difference between groups for lead I (W = 628.5, p = 0.002) was identified (Table 3). The most fixations were made in lead V1 and then the rhythm strip for both of the groups.

**Table 3.** Pairwise comparisons for each lead (Mann-Whitney U) with Bonferroni correction α = 0.004.

**ECG lead name**	W	p-value
I	628.5	0.002*
II	467.5	0.071
III	591	0.741
aVR	253	0.186
aVL	2021	0.702
aVF	1532.5	0.594
V1	3479.5	0.695
V2	12994	0.346
V3	5679.5	0.452
V4	675	0.017
V5	1294	0.022
V6	363	0.046
Rhythm strip (II)	6777	0.016

**Figure 8.**
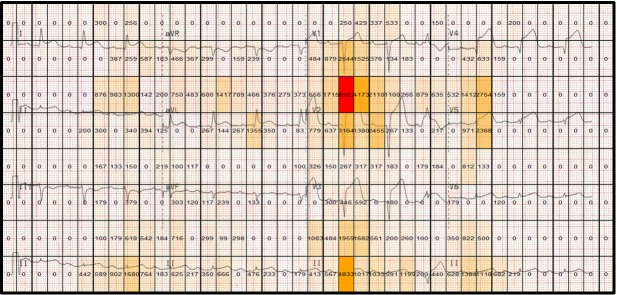

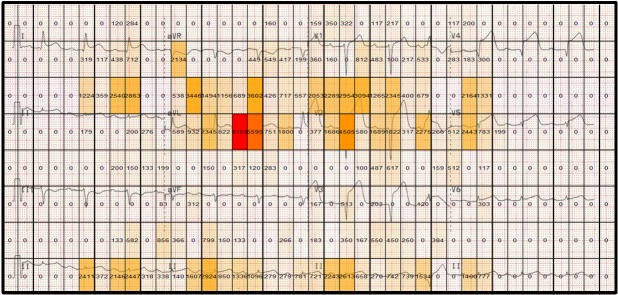
Heatmaps showing the total fixation duration in each grid cell for the anterolateral STEMI stimulus. (a) Correct group, (b) Incorrect group

Figure 8 highlights differences between the correct and incorrect groups for the anterolateral STEMI stimulus in relation to the dwell time (total fixation time) for each grid cell (displayed in each cell). The correct group has a greater dwell time in lead V1 and V2, which are two of the most important leads for providing clues to interpret this particular stimulus (ST segment elevation in the anterior and lateral leads). In contrast the incorrect group dwells mostly on the less useful lead (aVL). By segmenting the stimulus into equal sized regions and proving a numerical overlay on each cell, specific areas of stimuli can be more readily compared, with measurable differences between cells easily computed. This also provides an overview of the focus of attention made by both groups.

**Figure 9.**
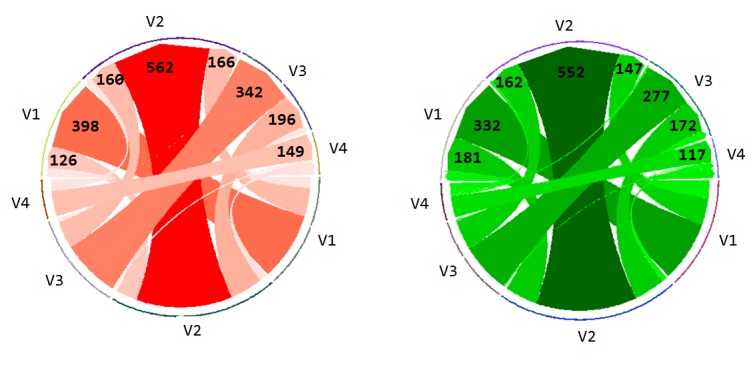
Chord diagrams representing the number of visual transitions from one lead to another (or within the same lead) for the incorrect (left) and correct (right) groups. The thicker the line the more transitions occurred. The arrow head displays the direction of the transitions.

Finally the transitions between the leads (V1-V4) presenting the most relevant salient information (highest degree of ST-segment elevation) are computed for both of the groups. Figure 9 shows the number of transitions from one lead to another or within the same lead itself. The number of transitions is represented by the thickness of the arrow, with the arrow point showing the direction of the transition (from - to). The actual number of transitions is also displayed on arrow heads. The incorrect group made a greater number of overall transitions (n=2307) than the correct group (n=2146).

## Discussion

Data-driven analysis can be challenging, especially when exploring factors such as accuracy, which can only be determined on a post hoc basis. The various visualizations applied to the data through this work provide useful information about and insights into the differences in visual behavior between these two groups. The “heart attack” stimulus is of special interest due to the clinical urgency of the condition and death by ischemic heart disease remaining the leading cause of mortality globally ( 23 ).

Overall we see a greater variability in the scanpaths between, rather than within, the two groups. When we consider differences in fixations on the leads of the ECG, we identify a significant difference between the accurate and inaccurate groups for lead I using a conservative approach. Lead I is not one of the leads showing the greatest degree of ST-segment elevation. It does, however, help the interpreter to see that there is elevation in the lateral leads as well as the anterior leads - leading to the conclusion that the interpretation should reflect lateral as well as anterior involvement. Comparing the leads on a pairwise basis may also be over simplistic; as the time spent viewing different leads may have an impact on time spent viewing subsequent leads. The heatmaps do, however, indicate that the correct group focuses more attentional resources on the lead showing the greatest degree of ST-segment elevation (the salient clue essential to identifying a heart attack).

The results of the analysis show large differences between the participants' individual scanpaths, which is indicative of differing search strategies. This difference could be attributable to the disparate backgrounds of the participants. There are many different methods of teaching ECG interpretation that vary in approach and duration ( 24 ). These methods also differ between countries and institutions as well as varying according to the medical discipline that the practitioner belongs to ( 25 ). Using a matrix to visualize the similarities/differences between the scanpaths with the Levenshtein distance is a helpful initial way of gaining a comparative overview of multiple participants in a study, and locating outliers who have markedly different or similar scanpaths. This can complement traditional methods, such as box plots.

An example of this is seen in the Levenshtein distance matrix (Figure 5). Here we can see participant 13 is a clear outlier and has a markedly different scanpath to all of the other participants in his group. This shows that metrics such as dwell time and fixation duration alone do not give us the whole picture with regard to behaviour and strategy. A richer understanding can be obtained by combining approaches to explore different aspects, such as temporal and sequential factors.

Scanpath analysis suffers from some limitations, including the issue of scanpath length, with very different lengths confounding alignment calculations ( 19 ). It should also be noted that visual behavior is very rich, and “naïve” scanpath analysis will not tell the whole story. Future work will focus on refining this approach, by considering visual transitions between leads, which is discussed in more detail in other work ( 26 ), and will also consider how factors such as accuracy of interpretation affect the results in greater detail.

The gridded heatmap visualizations serve a qualitative function, as visual differences in fixation duration can be quite striking. As the areas (cells) share the same size, direct comparison can be made quantitatively to focus on certain areas. Gridded AOIs also allow for analysis to take place in a content independent manner ( 27 ). The use of gridded AOIs and the segmentation approach used is consistent with the recommendations of ( 28 ) that AOIs margins should be predefined or based on data. In this case we can see that the fixations are clustered around smaller areas within the leads. This is consistent with the fact that practitioners need to measure changes in durations and morphologies of different parts of the ECG waveform in different conditions ( 29 ).

This is in keeping with previous work that demonstrates people tend to focus on some leads more than others ( 30 ). This indicates that participants were drawn toward specific features, possibly the lead or a component of the waveform that displays features of the ECG abnormality. This may also be the case regardless of making a correct or incorrect interpretation, as a participant may notice an abnormal feature without necessarily understanding its significance. Eye tracking data is frequently used to augment usability studies ( 2 ). The small sample sizes frequently used in usability studies coupled with the richness of eye-tracking data can make analysis of datasets such as the one used in this study challenging and often not amenable to traditional statistical approaches. The techniques described in this paper go some way toward providing a quantitative approach for exploration of this type of data, and we therefore anticipate they will have a scope wider than the ECG sub-domain, as they provide a means of understanding whether individuals are employing a systematic approach, or have some intrinsic similarity in their visual behavior.

### Conclusions and future work

The methods presented here offer a way of exploring and visualizing the visual behavior of practitioners viewing ECGs. They allow us to visualize differences in scanpaths that can indicate different search strategies, which may result from different training or experience. A weighted distance metric could also be introduced to incorporate the effect of time spent viewing the areas, as well as transitions between them. The techniques in this work provide a way of viewing the similarities and differences in multiple scanpaths and stimuli simultaneously, providing a quantifiable measure of difference without increasing visual complexity. The results of this study may be of future use in clinical practice, as differences in visual behavior may be used to identify potential failures to correctly interpreting ECGs that could be fed back to the practitioner in training scenarios.

### Ethics and Conflict of Interest

The authors declare that the contents of the article are in agreement with the ethics described in http://biblio.unibe.ch/portale/elibrary/BOP/jemr/ethics.html and that there is no conflict of interest regarding the publication of this paper.

### Acknowledgements

**EPSRC**: EP/K502947/1 and EP/L504877/1
